# Refusal to participate in research among hard-to-reach populations: The case of detained persons

**DOI:** 10.1371/journal.pone.0282083

**Published:** 2023-03-03

**Authors:** Stéphanie Baggio, Leonel Gonçalves, Patrick Heller, Hans Wolff, Laurent Gétaz

**Affiliations:** 1 Division of Prison Health, Geneva University Hospitals & University of Geneva, Geneva, Switzerland; 2 Institute of Primary Health Care (BIHAM), University of Bern, Bern, Switzerland; 3 Department of Psychiatry, Geneva University Hospitals, Geneva, Switzerland; 4 Division of Tropical and Humanitarian Medicine, Geneva University Hospitals, Geneva, Switzerland; University of North Carolina at Chapel Hill, UNITED STATES

## Abstract

Providing insights on refusal to participate in research is critical to achieve a better understanding of the non-response bias. Little is known on people who refused to participate, especially in hard-to-reach populations such as detained persons. This study investigated the potential non-response bias among detained persons, comparing participants who accepted or refused to sign a one-time general informed consent. We used data collected in a cross-sectional study primary designed to evaluate a one-time general informed consent for research. A total of 190 participants were included in the study (response rate = 84.7%). The main outcome was the acceptance to sign the informed consent, used as a proxy to evaluate non-response. We collected sociodemographic variables, health literacy, and self-reported clinical information. A total of 83.2% of the participants signed the informed consent. In the multivariable model after lasso selection and according to the relative bias, the most important predictors were the level of education (OR = 2.13, bias = 20.7%), health insurance status (OR = 2.04, bias = 7.8%), need of another study language (OR = 0.21, bias = 39.4%), health literacy (OR = 2.20, bias = 10.0%), and region of origin (not included in the lasso regression model, bias = 9.2%). Clinical characteristics were not significantly associated with the main outcome and had low relative biases (≤ 2.7%). Refusers were more likely to have social vulnerabilities than consenters, but clinical vulnerabilities were similar in both groups. The non-response bias probably occurred in this prison population. Therefore, efforts should be made to reach this vulnerable population, improve participation in research, and ensure a fair and equitable distribution of research benefits.

## Introduction

The non-response bias is a bias that occurs due to systematic differences between respondents and non-respondents. To better understand the non-response bias, insights on response rates and refusal to participate in research are needed, e.g., to assess whether participants are representative of the target population and to find out whether estimates are reliable. To date, little is known on people who refuse to participate, precisely because they decline participation. This is especially true for some hard-to-reach and vulnerable populations, such as detained persons.

Previous studies used late respondents as a proxy for non-respondents or compared respondents to non-respondents when information is available from the census population. For example, in their well-designed study in the substance use field, Studer et al. [1] concluded that non-respondents, late respondents, and respondents were significantly different from each other on substance use variables, with a non-negligible non-response bias. Other studies focusing on health-related topics, such as psychiatry [2, 3], suicide [4], chronic diseases [5], and sexually transmitted diseases [6, 7] obtained similar conclusions.

Overall, non-respondents appeared as more vulnerable than respondents. In the aforementioned studies, they displayed higher rates of substance use, psychiatric morbidity [1–4, 8], severe chronic and infectious diseases [5–7], and lower health literacy prevalence estimates [9]. In addition, non-respondents usually display specific socioeconomic characteristics. For instance, they are more likely to come from deprived backgrounds and low socioeconomic strata [7, 10–12].

Unfortunately, we know little about the non-response bias in detained populations, a vulnerable hard-to-reach population, with a severe burden of diseases and barriers to health care [13–16]. This study therefore aimed to investigate the potential non-response bias among detained persons, comparing participants who accepted to sign a one-time general informed consent to those who refused. The one-time informed consent was used as a proxy to understand the non-response bias, as information on detained persons who decline study participation is usually not available.

## Materials and methods

### Design and setting

We used data collected in a study primary designed to evaluate a one-time general informed consent for research [17]. The one-time general informed consent for research allows using routinely collected data from the hospital’s medical files [18].

The study had a cross-sectional design with a parallel randomization (allocation 1:1 for groups reading a paper version of the informed consent or watching a video, see link in S1 File). The study took place between December 2019 and December 2020 in the largest pre-trial Swiss prison (398 places), located in Geneva, in the French-speaking part of Switzerland, among male detained persons.

Participants provided oral consent to participate in the study, because the outcome of the main study was to provide a written one-time informed consent for research. Participants could refuse study participation. They were informed that they were free to participate and could refuse to participate or discontinue at any time, without any health care- or prison-related consequences. Participants received an incentive of CHF 20.- (~ 20€) for study participation (including those who did not signed the one-time informed consent). They were informed there was an incentive when they were invited to participate. The Geneva’s cantonal ethics committee approved the study protocol (no. 2019–01797), including the separate oral consent for study participation. Oral consent was registered on a separate file before participants’ randomization.

### Participants

A total of 228 adult men were invited to participate (oral consent), of which n = 193 accepted (response rate = 84.7%). Three participants dropped out before the end of the study (i.e., they did not sign the one-time informed consent and did not respond to the 15-minute questionnaire), which left a final sample of 190 participants. Among these 190 participants, 158 (83.2%) signed the one-time informed consent and 32 (16.8%) declined. Refusers nonetheless consented to study participation and completed the questionnaire. The only exclusion criterion was having severe acute psychiatric issues that did not allow informed consent.

### Procedures

A study member from the prison medical unit conducted data collection (enrollment of participants, consent process, and 15-minute questionnaire), supervised by SB and LG. The study was conducted independently from the prison authorities and the prison staff was not involved. Study participation was offered to eligible participants visiting the prison medical unit (about 75% of persons detained in the current prison), while they were in the waiting room.

Participants either read a booklet or saw a video (the experimental condition in the primary study) and were invited to sign the informed consent (see S1 File). Then, they all completed a 15-minute face-to-face questionnaire (including participants who refused to sign the informed consent). Participants first answered questions about the informed consent (questions not included in this study [17]) and then questions about sociodemographic and clinical variables.

The informed consent and questionnaire were available in the ten most common languages spoken in the prison: Albanian, Arabic, English, French, Georgian, German, Italian, Portuguese, Romanian, Russian, and Spanish.

### Measures

#### Acceptance to sign the informed consent

Participants could either agree (consenters) or decline (refusers) to sign the informed consent (legal Swiss document, see: https://swissethics.ch/en/templates/studieninformationen-und-einwilligungen). This variable was used as a proxy to assess the non-response bias.

#### Sociodemographic variables

Participants provided information on age, region of origin (Switzerland or European Union [EU] versus other countries), level of education (primary versus secondary/tertiary level of education), legal status of residence in Switzerland (yes/no), and health insurance status (having an insurance or not, which is mandatory in Switzerland). We also registered the language participants selected for their participation in the study (French versus other) and whether they would have preferred to answer in another language (yes/no).

#### Health literacy

Health literacy was assessed using the three-question Short Test of Functional Health Literacy (S-TOFHLA) [19]. As the scale was negatively skewed, we recoded data in two categories: High versus low/moderate health literacy.

#### Clinical information

Participants self-reported the presence of psychiatric disorders and somatic illnesses.

### Statistical analyses

We first computed descriptive statistics for all variables (percentages and frequencies for binary variables, means and standard deviations for continuous variables). We also computed the relative bias as indicated in Studer et al. [1]:

$$Relative\;bias=\left(\frac{N_{nr}}{N_{tot}}\times \left({\overset{-}{Y}}_{nr}-{\overset{-}{Y}}_{r}\right)\right)÷{\overset{-}{Y}}_{tot}$$

Where $\frac{N_{nr}}{N_{tot}}$ is the non-response rate, ${\overset{-}{Y}}_{nr}$ the mean (or proportion) for refusers, ${\overset{-}{Y}}_{r}$ the mean (or proportion) for consenters, and ${\overset{-}{Y}}_{tot}$ the mean for the whole sample. We reported absolute values, as the sign did not affect the magnitude of the bias and was not of interest for our study. Second, we assessed the bivariate associations between acceptance to sign the informed consent and participants’ characteristics (i.e., sociodemographic variables, health literacy, and clinical information) using simple logistic regressions. Third, we performed a multivariable logistic regression including all covariates using least absolute shrinkage and selection operator (lasso) to select the most important predictors of acceptance to sign the informed consent. We also controlled for the study intervention (randomization in the booklet or video groups). Lasso is a reliable way to select variables, enhancing prediction accuracy and interpretability of the model [20], without the limitations of stepwise regression [21]. In this study, lasso selection was used in the multivariable model because the sample size was rather small in comparison with the number of covariables. No replacement was made for missing values (see Table 1 for detailed information on missing values). Statistical significance was set at p < .05. The analyses were performed with Stata 17.

**Table 1:** Descriptive characteristics of consenters and refusers.

	Overall	Consenters	Refusers	Missing values	Relative bias
	n = 190	n = 158	n = 32		
Age (mean, sd)	35.0 (11.8)	35.1 (12.0)	34.5 (10.5)	1	-0.3%
Region of origin (%, n)					
Other countries	63.6 (119)	60.3 (94)	80.7 (25)	3	5.3%
CH and EU	36.7 (68)	39.7 (62)	19.3 (6)		-9.2%
Level of education (%, n)					
Primary	12.8 (24)	10.1 (16)	26.7 (8)	2	20.7%
Secondary/tertiary	87.2 (164)	89.9 (142)	73.3 (22)		-3.0%
Legal status in CH (%, n)					
No	56.2 (104)	54.2 (84)	66.7 (20)	5	3.6%
Yes	43.8 (81)	45.8 (71)	33.3 (10)		-4.6%
Health insurance (%, n)					
No	50.0 (94)	46.2 (73)	70.0 (21)	2	7.6%
Yes	50.0 (94)	53.8 (85)	30.0 (9)		-7.8%
Study language (%, n)					
Other than French	42.1 (80)	39.2 (62)	56.3 (18)	0	6.8%
French	57.9 (110)	60.8 (96)	43.7 (14)		-5.0%
Needed another language (%, n)					
No	94.7 (180)	96.8 (153)	84.4 (27)	0	-2.2%
Yes	5.3 (10)	3.2 (5)	15.6 (5)		39.4%
Health literacy (%, n)					
Low/moderate	41.5 (78)	37.3 (59)	63.3 (19)	2	10.0%
High	58.5 (110)	62.7 (99)	36.7 (11)		-7.1%
Any somatic illness (%, n)					
No	60.3 (114)	61.4 (97)	54.8 (17)	1	-1.8%
Yes	39.7 (75)	38.6 (61)	45.2 (14)		2.7%
Any psychiatric disease (%, n)					
No	79.9 (151)	79.8 (126)	80.7 (25)	1	0.2%
Yes	20.1 (38)	20.2 (32)	19.3 (6)		-0.7%
Randomization (%, n)^1^					
Booklet group	50.0 (95)	48.7 (77)	56.3 (18)	0	2.6%
Video group	50.0 (95)	51.3 (81)	43.7 (14)		-2.6%

Sd: standard deviation, CH: Switzerland, EU: European Union.

E.g., relative bias for need of another language (yes): ((32/190)*(.156-.032))/.053. For variables with missing values, the response rate (first part of the formula) was computed according to real numbers of the corresponding variable (e.g., for primary level of education: ((30/188)*(.267-.101))/.128. The sign of the relative risk indicates whether the mean/proportion is lower for the consenters compared to the refusers (positive sign) or higher for the consenters compared to the refusers (negative sign). It does not affect the magnitude of the relative bias.

^1^ Randomization with allocation 1:1 in the booklet or video group.

## Results

Descriptive statistics are reported in Table 1. Participants were on average 35.0 ± 11.8 years. Most of them came from countries outside the EU (63.6%), had a secondary or tertiary level of education (87.2%), and had no legal status in Switzerland (56.2%). Half of them did not have a health insurance (50.0%). They reported a high health literacy (58.5%) and few health conditions (somatic: 39.7%, psychiatric: 20.1%). Most participants chose French (57.9%) and 5.3% reported the need for another language than the ten available for the present study. No detained persons declined participation because of language difficulties. Regarding the outcome variable, a total of 83.2% signed the informed consent, whereas 16.8% declined.

We found the highest relative biases for the need of another language (39.4% for the group who answered “yes”), level of education (20.7% for the group with a primary level of education), health literacy (10.0% for the group with a low level of health literacy), region of origin (|9.2|% for the group from Switzerland or UE), health insurance (|7.8|% for the group having a health insurance), and study language (6.8% for the group that chose other languages than French).

Associations between acceptance to sign the consent and covariates are presented in Table 2. In bivariate models, consenters were more likely to come from Switzerland and EU (odd ratio [OR] = 2.75, p = .036), to have a secondary or tertiary level of education (OR = 3.23, p = .017,), to have a health insurance (OR = 2.72, p = .020), and to have a high health literacy (OR = 2.90, p = .010). They were less likely to need another study language (OR = 0.18, p = .009). There were no differences between consenters and refusers for age (p = .778), legal status in Switzerland (p = .211), and health problems (somatic: p = .496, psychiatric: p = .909). In the multivariate model after lasso selection, statistically significant predictors were level of education (OR = 2.13), health insurance status (OR = 2.04), need of another study language (OR = 0.21), and health literacy (OR = 2.20).

**Table 2:** Associations between acceptance to sign the consent and covariates.

	Logistic regression			Lasso selection^1^
	OR	p	95% CI	OR
Age	1.01	.778	0.97; 1.04	-
Region of origin (ref. other countries)	2.75	.036	1.07; 7.09	-
Level of education (ref. primary)	3.23	.017	1.24; 8.43	2.13
Legal status in CH (ref. no)	1.69	.211	0.74; 3.85	-
Health insurance (ref. no)	2.72	.020	1.17; 6.30	2.04
Study language (ref. other than French)	1.99	.079	0.92; 4.29	-
Needed another language (ref. no)	0.18	.009	0.05; 0.65	0.21
Health literacy (ref. low/moderate)	2.90	.010	1.29; 6.51	2.20
Any somatic illness (ref. no)	0.76	.496	0.35; 1.66	-
Any psychiatric disease (ref. no)	1.06	.909	0.40; 2.80	-
Randomization (ref. booklet)	1.35	.439	0.63; 2.91	-

CI: confidence intervals; OR: odd-ratio.

^1^ Lasso selection after logistic regression.

## Discussion

This study investigated the potential non-response bias among detained persons. For this purpose, we tested whether detained persons who refused to sign a one-time general informed consent were different from those who accepted.

Study findings showed that participants who declined participation displayed specific features compared to those who accepted. They were more likely to have social vulnerabilities, including educational barriers (low level of education), language and cultural barriers (especially not speaking one of the ten most common languages in the prison, but also being a migrant from outside the EU, and not speaking the region’s language), and health care-related barriers (low level of health literacy and no health insurance). These findings are in line with previous studies reporting that non-respondents were more likely to come from low socioeconomic areas, including low educational levels and migration backgrounds [7, 10–12] and that non-response bias is related to health literacy [9].

Detained persons are a population with severe social vulnerabilities, likely to come from deprived backgrounds [13]. Taken together, our findings suggested that detained persons who refused to participate in research were a more vulnerable subgroup of this already disadvantaged population.

To date, few studies investigated the association between non-response and health insurance coverage. In the USA, a study showed that non-respondents were less likely to have a health insurance coverage compared to respondents [22]. Other study findings suggested that health care use was lower in non-respondents, among people having a health insurance coverage [23]. Switzerland has a compulsory universal health care coverage, paid by the individuals. State subsidies are available to ensure that everyone can afford basic health insurance. However, in our study, half of the participants did not have a health insurance.

Another important study finding was that refusers did not display clinical vulnerability, with similar proportions of somatic and psychiatric health problems. This result was inconsistent with previous research findings in the general population, which concluded that non-respondents had more psychiatric morbidity and somatic illnesses [1, 2, 4–7]. Potential explanations of this study findings are related to the type of population and access to health care. First, the prison population might be younger than population-based studies. Therefore, detained persons are less likely to have some severe somatic chronic diseases that occur in middle age. Second, detained persons often lack access to (primary) health care before and during detention and may therefore have underdiagnosed and undertreated somatic and psychiatric diseases [24]. They are thus unaware of potential somatic or psychiatric issues. Indeed, in our sample, only 20.1% of participants self-reported mental health problems, which is probably an underestimation of the true prevalence rate of mental health problems in the prison. In a previous meta-analysis, 39.8% to 49.2% of detained persons were identified as suffering from mental illnesses [14–16].

Refusal to participate in health research is a well-known threat for study validity, but not only. It is also likely to reinforce existing health inequalities, as refusers display different characteristics and may have different study outcomes. Since refusal to participate is more frequent in vulnerable people and hard-to-reach populations, with high health needs [25] and lack of access to primary health care [26], they may be excluded from research benefits [27]. For example, the exclusion of vulnerable subgroups from clinical trials (e.g., elderly) limit the generalizability of findings, with insufficient data about positive or negative effects of treatments [28]. Such exclusion may hinder access to new treatment and high-quality care [27, 28]. Including vulnerable populations in research is critical for evidence-based medicine: It would enhance external validity, provide a fair access to research benefits, and improve understanding of vulnerabilities [25, 29].

Previous studies concluded that the non-response bias is less critical when basic sociodemographic variables of responders and non-responders are similar or when the proportion of responders exceeds 70% [30]. Our study findings suggested that with similar age and a response rate of more than 85%, non-response bias might still exist. The non-response bias should be reduced, with awareness that simple rules may not apply and efforts to encourage study participation. To improve study participation, strategies such as interview incentives, community contacts, use of different recruitment channels, and promote trusting relationships could be used [12, 25]. Guidelines are also available to improve informed consent, such as the “teach-to-goal” consent [29]. It helps to achieve a voluntary and truly informed consent.

This study had some limitations. First, the study took place at the prison medical unit. Detained persons who did not seek for health care were therefore not included (~25% of detained persons). This subgroup of detained persons was probably heterogenous, including people who did not need health care, but also people with specific vulnerabilities (e.g., language barriers, anosognosia). Second, some eligible participants declined study participation and were therefore not included in the “refusal” group (15.3%). These non-respondents could have different characteristics and our results should therefore be interpreted cautiously. The one-time informed consent was used as a proxy of non-response bias, but further studies should include detained persons who completely refuse to participate. Third, the study had a small sample size in some subgroups (i.e., participants without a legal status in Switzerland), with a potential lack of power. Fourth, data were collected among detained men. To avoid an increased gender bias in research [31], future studies should include women, even if detained persons are mostly men. Fifth, we relied on self-reports, which limited the reliability of clinical information. Use of census, administrative, and medical encounter data would be helpful to describe consenters and refusers more accurately. In addition, the reliability of self-reported information for refusers was questionable. Sixth, the S-TOFHLA assesses ability of individuals to read and understand health-related information, but it does not assess numeracy, which is an important dimension for health literacy [32]. Further studies should include a more complete range of measures of health literacy. Seventh, given important differences in sociopolitical contexts, penal systems, and prison populations, these results cannot be generalized outside Switzerland.

To conclude, the non-response bias probably occurs in prison populations, as it is the case in the general population. Detained persons who declined research participation were more likely to have social vulnerabilities. Therefore, efforts should be made to reach this vulnerable population, minimize non-response, and ensure a fair and equitable distribution of research benefits [12].

## Supporting information

S1 File (PDF)
