# Supplementary material for: Refusal to participate in research among hard-to-reach populations: The case of detained persons
Source: PLoS One. 2023 Mar 3;18(3):e0282083. doi: 10.1371/journal.pone.0282083 (PMC9983841; doi:10.1371/journal.pone.0282083)
Supplement: S1 File — (PDF) [file pone.0282083.s001.pdf]

Video link:

https://vimeo.com/654887207/fc26d1e957

**Introducing the study to eligible participants:**
Good morning, sir. Would you agree to give us 20 minutes for a medical research project and answer a few questions? If you accept, you will receive 20 francs to make purchases in the prison canteen.
This study is about consent for research: we will ask you to reuse your medical data for research. First you will see a video / receive a brochure, on consent then you will tell us if you agree to sign the consent for the reuse of your data. Finally, you will be asked some questions about yourself and what you thought of the video / brochure.
